# Factors associated with voluntary HIV counseling and testing among young students engaging in casual sexual activity: a cross-sectional study from Eastern China

**DOI:** 10.1186/s12889-024-18562-2

**Published:** 2024-04-22

**Authors:** Zhongrong Yang, Wanjun Chen, Weiyong Chen, Qiaoqin Ma, Hui Wang, Tingting Jiang, Meihua Jin, Xin Zhou

**Affiliations:** 1 Huzhou Center for Disease Control and Prevention, Zhejiang province, 313000 Huzhou, China; 2 Department of HIV/STD control and prevention, Zhejiang Provincial Center for Disease Control and Prevention, No.3399, Binsheng Road, 310051 Hangzhou, Zhejiang province China

**Keywords:** HIV, VCT, Cross-sectional study, AIDS, Casual sexual activity, Associated factors, Student, China

## Abstract

**Objectives:**

To investigate the factors associated with voluntary HIV counseling and testing (VCT) among young students engaging in casual sexual activity and to establish a scientific rationale for developing targeted intervention strategies for preventing HIV/AIDS in this population.

**Methods:**

Stratified cluster sampling was used to conduct a survey using questionnaires to collect demographic and behavioral information for statistical analysis.

**Results:**

Data from 611 young students, who reported engaging in casual sexual activity, were included in the statistical analysis. Among these, 68 (11.13%) students underwent the VCT. Among young students who engaged in casual sexual activity, those who were non-Zhejiang residents (adjusted odds ratio [*aOR*]: 2.11; 95% Confidence Interval [*CI*]: 1.17–3.80), those who had received AIDS-themed lectures or health education courses from the school in the past year (*aOR* = 3.96, 95% *CI* = 1.49–10.50), those who had received HIV risk self-assessment conducted by the school in the past year (*aOR* = 2.31, 95% *CI* = 1.17–4.59), and those who had engaged in commercial sex activity in the past year (*aOR* = 1.98, 95% *CI* = 1.07–3.66) were more inclined to have undergone VCT. Male students (*aOR* = 0.37, 95% *CI* = 0.18–0.77) and those who used condoms consistently during casual sexual activity (*aOR* = 0.45, 95% *CI* = 0.21–0.97) were less likely to undergo VCT.

**Conclusion:**

Casual sexual activity was relatively prevalent among young students, posing a potential risk for HIV transmission. These findings will be instrumental in the development more effective HIV prevention and control strategies for young students. Additionally, it highlights the necessity of promoting and popularizing VCT among young students without Zhejiang province residency, who are involved in commercial sexual activity, and/or those who lacking HIV education. Moreover, additional research and implementation of refined HIV behavioral interventions specifically tailored to young students are necessary to enhance their awareness and knowledge of HIV prevention.

## Introduction

Acquired immunodeficiency syndrome (AIDS), caused by the human immunodeficiency virus (HIV), poses a significant threat to public health as an infectious disease and has emerged as a major burden on healthcare systems and is a pressing global public health concern [1, 2]. HIV, specifically targets CD4^+^ T lymphocytes, which play a crucial role in immune function and protecting against attacks on the human immune system. HIV infection results in the widespread destruction of these cells, gradually impairing the immune system and rendering the body susceptible to various other infections. Without treatment, this ultimately leads to systemic organ failure, which often results in death [3].

Currently, significant progress has been made in global HIV/AIDS prevention and control efforts, leading to a decline in mortality and infection rates [4, 5]. The Joint United Nations Programme on HIV and AIDS (i.e., “UNAIDS”) launched an ambitious target of “95-95-95”; 95% of people living with HIV know their infection status, 95% of diagnosed individuals receive sustained antiretroviral therapy, and 95% of treated individuals achieve viral suppression [6]. The Chinese government places significant emphasis on HIV/AIDS prevention and control, as evidenced by the formulation, implementation, and promotion of a comprehensive range of policies, measures, nation-wide HIV testing and antiretroviral therapy. Simultaneously, China is enhancing the dissemination of knowledge regarding HIV/AIDS prevention and control to raise public awareness for self-protection. Nevertheless, HIV/AIDS prevention and control efforts at both the global and national levels in China encounter several challenges such as insufficient coverage of viral testing, treatment monitoring, and drug resistance. As such, researchers and medical institutions must make additional efforts to implement and enhance prevention and control measures to establish a solid foundation for eventual eradication of HIV/AIDS.

In recent years, there has been growing concern about the increasing severity of the HIV epidemic among young students [7–9]. The phrase “young people” encompasses individuals between 15 and 24 years of age. Globally, approximately 37.7 million individuals live with HIV/AIDS, approximately 90% of whom are young adults [10]. In 2017, the number of newly diagnosed cases of HIV infection among Chinese university students was more than ten times higher than in 2006, with 3077 cases reported in 2017 compared to 242 cases in 2006, and the annual growth rate during this period ranged from 30 to 50% [9]. In recent years, the number of newly reported HIV/AIDS cases among young students in China has remained stable at approximately 3,000 annually [11, 12]. Additionally, around 100 new cases of HIV/AIDS have been diagnosed among college students in Zhejiang Province in recent years [9]. Considering their active sexual behavior, young students who engage in sexual activity may be more vulnerable to sexually transmitted diseases (STDs) including HIV [13, 14]. Youth, being the future “pillars of society”, have the potential to contribute to the country’s development by receiving high-quality education and training and becoming professionals in various fields. Therefore, preventing the transmission of HIV among young students is indispensable to their well-being and to society as a whole.

The primary goal of HIV voluntary counseling and testing (VCT) is to encourage and assist individuals in comprehending their HIV infection status and implement appropriate intervention measures through the provision of HIV testing and counseling services [15]. There are many qualitative reasons for participating in VCT, including individual attention, community mobilization, sexual health services, and awareness of self-protection [15, 16]. Research has demonstrated the positive impact of VCT on HIV prevention and control among young students by enhancing their knowledge and awareness of HIV and offering a confidential, reliable, and convenient testing approach, which ultimately increases the chances of early detection, diagnosis, and treatment of individuals who have contracted HIV [17]. Infected individuals can benefit from VCT through personalized prevention and treatment strategies aimed at reducing the risk for HIV transmission. HIV-negative individuals can enhance their self-protection awareness through counseling, effectively reducing the risk for HIV infection.

To effectively reduce HIV transmission among young students, it is important to implement measures to strengthen health education, promote shifts in social attitudes, and ensure the provision of accessible and affordable VCT services. Currently, there is a limited body of research exploring the factors associated with VCT among young students who engage in casual sexual behavior(s). Casual sexual activities are common among young students, yet there is limited research specifically looking at VCT uptake among this population. Understanding the factors influencing VCT uptake among students engaging in casual sex is crucial for developing targeted interventions to promote HIV testing and reduce transmission. To address this research gap, a cross-sectional survey was conducted in Zhejiang Province, eastern China, to investigate the factors related to VCT uptake among university students engaging in casual sexual activity. This will not only help in strengthening the motivation for our research but also provide a foundation for future studies and public health initiatives aimed at improving HIV testing rates and promoting safer sexual practices among this vulnerable population.

## Materials and methods

### Sampling method and participants

The present study used a cross-sectional survey design to investigate factors associated with VCT among college students from 13 universities in 11 districts and municipalities in Zhejiang Province between October and November, 2018. Among these, 3 universities were located in Hangzhou, and each of the remaining 10 cities had 1 university. Universities were selected based on recommendations from local disease control centers. Sample populations were collected using a stratified cluster sampling method. Three departments were randomly selected from each university using a random number table. Subsequently, the selected departments were further divided into four levels based on the students’ academic year (1st to 4th year). Finally, classes were chosen randomly from each level using a random number table. Data collection involved 1241 classes with 31,674 participating students.

### Ethical statement

This study protocol was approved by ethics committee of the Zhejiang Provincial Center for Disease Control and Prevention, and this study complied with the declaration of Helsinki and all methods were performed in accordance with the relevant guidelines and regulations of the ethics committee of the Zhejiang Provincial Center for Disease Control and Prevention (No.2018-036). All the participants provided written informed consent.

### Questionnaire design

The questionnaire was developed by reviewing the domestic and international literature [18, 19], engaging in discussions with the research team, and conducting a preliminary survey of students in a school class. The questionnaire primarily addresses demographic factors, knowledge of HIV prevention and treatment, attitudes toward sex, participation in interventions, and self-confidence in condom usage. Knowledge-based questions in the survey queried statements such as “Whether it can be judged by appearance that a person is infected with HIV” and “Whether it is necessary to actively seek VCT after engaging in high-risk sexual behavior”. Casual sex was defined as the sexual activity that the participant had engaged in with other males or females in the past year, apart from their fixed boyfriend or girlfriend. Commercial sex refers to sexual activity involving monetary transactions.

The effectiveness of measuring condom use involved 3 questions: level of confidence in discussing condom use with sexual partners before engaging in sexual activity; the level of confidence in abstaining from sex or engaging in sexual activity without using a condom, and level of confidence in preparing a condom before engaging in sexual activity. Each question offered respondents 5 options for their confidence levels: very confident, quite confident, confident, not confident, very unconfident, which corresponding scores of 3, 2, 1, 0, and − 1, respectively. The measurement scores were categorized as ≤ 4 or below 5–8, and 9. As per the assessment, the Cronbach’s alpha coefficient for this measurement was determined to be 0.794.

### Data collection

This study used a cross-sectional survey design. Students within the school were organized and assembled by teachers, who used their cellphones to scan quick response (i.e., “QR”) codes and completed online electronic surveys. Students not attending the school received a survey link and were instructed to complete it independently based on the instructions provided. Study participants included college students who self-reported engaging in casual sexual activity. There were no statistically significant differences in basic characteristics, such as age, gender, grade, province of household registration, hometown of origin, monthly living expenses, and family relationship, between the excluded population and the participants included in the study. The study participants were categorized into VCT and non-VCT groups.

### Household registration system in China

Household registration, known as “hukou,” denotes the officially registered residence documented by the public security authorities and specified in the resident’s household registration booklet and identity card. In line with the chinese household registration regulations and the prevailing household registration management system, newborns must be registered for birth within one month of their arrival by the household head, relatives, or caretakers at the local household registration office where the infant resides. The allocation of urban fundamental public services, encompassing compulsory education, employment assistance, basic pensions, primary healthcare, and housing security, among other services, is determined by the population figures of the hukou region in different areas. Discrepancies may exist across regions, subject to meeting the national minimum guarantee standards. Given potential changes in college students’ hukou due to residential relocations post-enrollment, this study defines the household registration based on the province of registration before university admission.

### Quality control

The investigators consisted of professionals from the local disease prevention and control center and counselors from the surveyed classes at various universities. They received standardized training and completed anonymous surveys using a standardized questionnaire. Before the study, the investigators explained the purpose, significance, methods, and privacy protection policy to the research participants, with this information also included in the introduction of the survey questionnaire. The research participants were informed that the investigation aimed to develop strategies for preventing HIV and other STDs among students. Participants were assured of anonymity and that only group data would be analyzed, at the exclusion of any individual identifiable data.

### Data analysis

Data analysis was performed using SPSS version 23.0 (IBM Corporation, Armonk, NY, USA). Various variables, including age, sex, grade, province of household registration, source of origin hometown, monthly living expenses, family relationships, sexual attitudes, HIV/AIDS prevention and control knowledge, and sexual behavior characteristics, are expressed as composition ratios or rates. The chi-squared test was used to compare the demographic characteristics of the participants in the VCT. The independent variables included demographic factors, sexual attitudes, knowledge of prevention and control, acceptance of the intervention, and self-efficacy in condom use. Furthermore, a single-factor logistic regression method was used to analyze factors associated with VCT among the participants. Variables with *P* < 0.2 were incorporated into the model for multivariable logistic regression analysis, ensuring statistical significance. Differences with *P* < 0.05 were considered to be statically significant.

## Results

### Demographic characteristics of participants

**Fig. 1:**
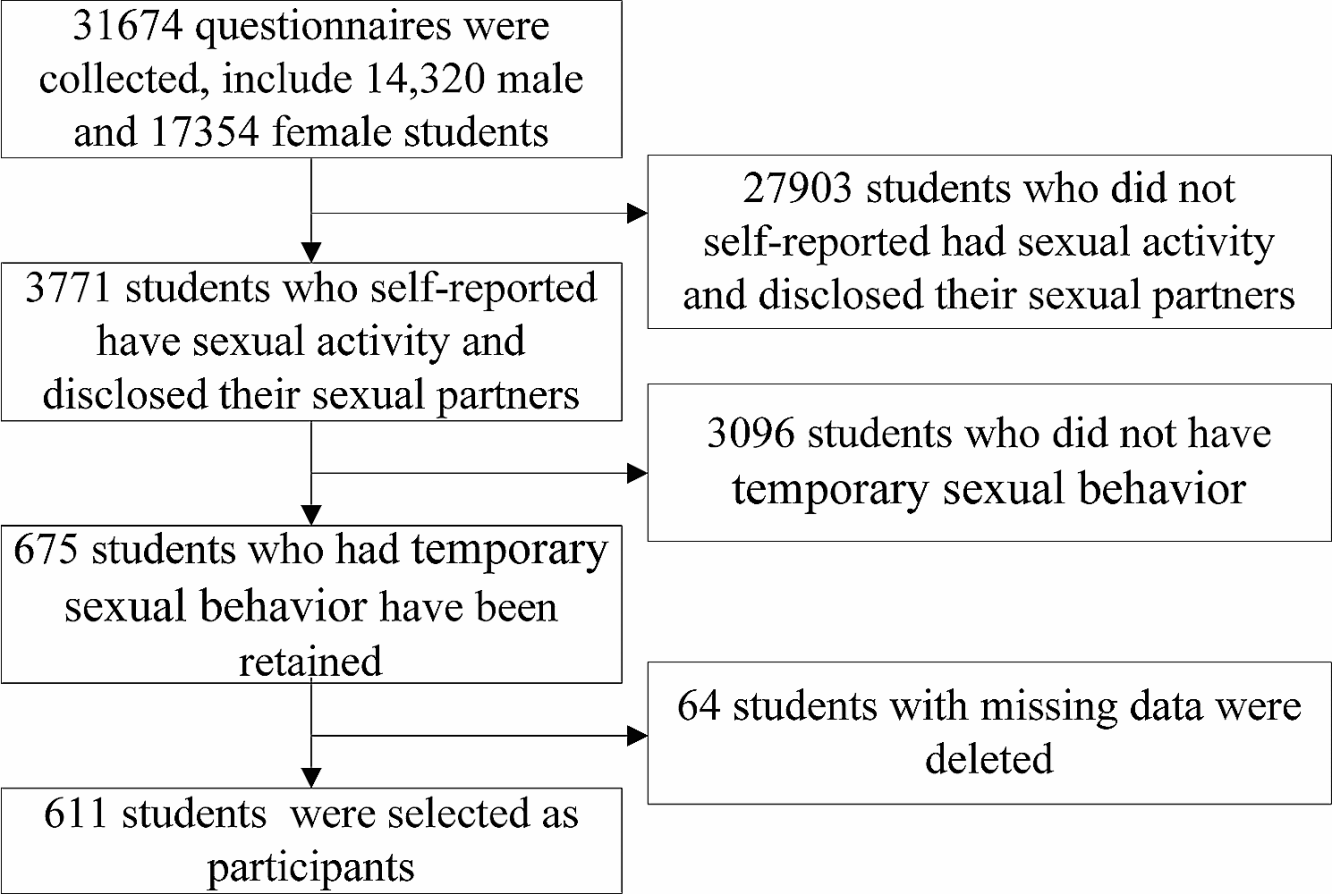
The flowchart for the inclusion and exclusion process

The study included a sample of university students, consisting of 14,320 males and 17,354 females. In total, 3,771 individuals reported engaging in sexual activity and informed their sexual partners. Among these, 675 (17.90% sexually active students) reported engaging in casual sex. Of these, 64 individuals with missing data were excluded, leaving 611 students who were selected for the analysis, as shown in Fig. 1. Among the participants who engaged in casual sex, 68 (11.13%) underwent VCT, and had a mean (± SD) age of 20.34 ± 1.98 years. The remaining 543 individuals, comprising 88.87% of the sample, did not undergo VCT, and had an average age of 20.07 ± 1.45 years. Statistically significant differences (i.e., *P* < 0.05) were observed between the VCT and non-VCT groups regarding sex and grade, whereas no statistically significant differences (i.e., *P* ≥ 0.05) were found in terms of age, province of household registration, hometown of origin, monthly living expenses, and family relationships (Table 1).

**Table 1:** Demographic characteristics of participants

	VCT group(*n* = 68, %)	Non-VCT group(*n* = 543, %)	χ^2^	*P*
**Age (yrs)**	20.34 ± 1.98	20.07 ± 1.45		
Less than or equal to 19	22(32.4)	185(34.1)	1.617	0.446
20–21	33(48.5)	285(52.5)		
Greater than or equal to 22	13(19.1)	73(13.4)		
**Gender**			4.681	0.030
Female	15(22.1)	68(12.5)		
Male	53(77.9)	475(87.5)		
**Grade**			8.488	0.037
Freshman	9(13.2)	111(20.4)		
Sophomore	34(50.0)	178(32.8)		
Junior	20(29.4)	187(34.4)		
Senior	5(7.4)	67(12.3)		
**Province of household registration**			3.170	0.075
Zhejiang province	43(63.2)	399(73.5)		
Non-Zhejiang province	25(36.8)	144(26.5)		
**Hometown of origin**			0.002	0.965
Rural area	32(47.1)	254(46.8)		
Town/City	36(52.9)	289(53.2)		
**Monthly living expenses (CNY** ^*^ **)**			2.979	0.226
≦ 1000	26(38.2)	159(29.3)		
1001–1500	21(30.9)	165(30.4)		
≧ 1501	21(30.9)	219(40.3)		
**Family relationship**			0.938	0.333
Harmonious	54(79.4)	418(77.0)		
General/disharmonious	14(20.6)	125(23.0)		

^*^CNY, Chinese Yuan

### Univariable analysis of factors associated with VCT among the participants

Results of univariable analysis (Table 2) revealed that participants who had received AIDS-themed lectures or health education courses from school in the past year (crude odds ratio [*cOR*] 3.75), those who had received HIV risk self-assessment conducted by school in the past year (*cOR* = 3.52), those who had engaged in sexual activity with a steady partner in the past year (*cOR* = 3.04), and those who had engaged in commercial sex activity in the past year (*cOR = 3.24*) were more likely to have undergone VCT. Participants who believed that “Whether it can be judged by appearance that a person is infected with HIV” (*cOR* = 0.50), and those who used condoms consistently or occasionally during casual sexual activity (*cOR* = 0.28 and 0.41, respectively) were less likely to have undergone VCT.

**Table 2:** Analysis of factors associated with VCT among the participants

Variables	VCT group(*n* = 68)		Non-VCT group(*n* = 543)		Univariable analysis			Multivariable analysis	
	n (%)		n (%)		cOR (95% CI)	*P*		aOR (95% CI)	*P*
**Gender**									
Female	16(13.6)		76(12.7)		Ref.1			Ref.1	
Male	59(78.7)		524(87.3)		0.51(0.27–0.95)	0.033		0.37(0.18–0.77)	0.008
**Grade**									
Freshman	10(13.3)		127(21.2)		Ref.1			Ref.1	
Sophomore	36(48.0)		199(33.2)		0.56(0.31–1.01)	0.054		0.60(0.32–1.14)	0.119
Junior	24(32.0)		199(33.2)		0.39(0.15–1.04)	0.060		0.51(0.18–1.47)	0.211
Senior	5(6.7)		75(12.5)		0.42(0.20–0.92)	0.030		0.46(0.20–1.07)	0.070
**Province of household registration**									
Zhejiang province	47(63.5)		444(74.0)		Ref.1			Ref.1	
Non-Zhejiang province	27(36.5)		156(26.0)		1.61(0.95–2.73)	0.077		2.11(1.17–3.81)	0.013
**Whether it can be judged by appearance that a person is infected with HIV?**									
Wrong/don’t know	26(38.2)		128(23.6)		Ref.1			Ref.1	
Correct	42(61.8)		415(76.4)		0.50(0.29–0.85)	0.010		1.04(0.55–1.96)	0.902
**Whether it is necessary to actively seek VCT after engaging in high-risk sexual behavior** ^**#**^									
Wrong/don’t know	8(11.8)		50(9.2)		Ref.1			——	
Correct	60(88.2)		493(90.8)		0.76(0.34–1.68)	0.499		——	
**Whether received AIDS-themed lectures or health education courses from the school in the past year**									
No	8(11.8)		181(33.3)		Ref.1			Ref.1	
Yes	60(88.2)		362(66.7)		3.75(1.76–8.01)	0.001		3.96(1.49–10.50)	0.006
**Whether received an HIV risk self-assessment conducted by the school in the past year**									
No	17(25.0)		293(54.0)		Ref.1			Ref.1	
Yes	51(75.0)		250(46.0)		3.52(1.98–6.24)	0.000		2.31(1.17–4.59)	0.016
**Whether learned about HIV/AIDS through the school network in the past year**									
No	13(19.1)		148(27.3)		Ref.1			Ref.1	
Yes	55(80.9)		395(72.7)		1.59(0.84–2.99)	0.154		0.45(0.19–1.07)	0.070
**Will you accept one night stand?**									
Don’t accept/don’t know	21(30.9)		124(22.8)		Ref.1			Ref.1	
Yes	47(69.1)		419(77.2)		0.66(0.38–1.15)	0.144		0.63(0.34–1.19)	0.156
**Do you accept commercial sex?**									
Don’t accept/don’t know	26(38.2)		253(46.6)		Ref.1			——	
Yes	42(61.8)		290(53.4)		1.41(0.84–2.36)	0.211		——	
**Casual partner types**									
School student	45(67.2)		343(64.0)		Ref.1			——	
Non-student	22(32.8)		193(36.0)		0.87(0.51–1.49)	0.610		——	
**Condom use with casual sexual partners**									
Never used	28(41.2)		106(19.5)		Ref.1			Ref.1	
Occasional used	24(35.3)		224(41.3)		0.41(0.22–0.73)	0.003		0.56(0.28–1.10)	0.090
Every time used	16(23.5)		213(34.9)		0.28(0.15–0.55)	0.000		0.45(0.21–0.97)	0.041
**Have you had engaged in sexual activity with a steady partner in the past year**									
No	12(17.6)		179(33.0)		Ref.1			Ref.1	
Yes	56(82.4)		364(67.0)		3.04(1.82–5.08)	0.000		1.87(0.91–3.83)	0.087
**Whether had engaged in commercial sex in the past year**									
No	31(45.6)		390(71.8)		Ref.1			Ref.1	
Yes	37(54.4)		153(28.2)		3.24(1.95–5.37)	0.000		1.98(1.07–3.66)	0.029
**Measurement of self-efficacy in condom use (scores)**									
Less than or equal to 4	16(23.5)		137(25.2)		Ref.1			Ref.1	
5–8	13(19.1)		175(32.2)		0.64(0.30–1.37)	0.247		0.75(0.32–1.73)	0.494
9	39(57.4)		231(42.5)		1.45(0.78–2.69)	0.243		1.48(0.72–3.10)	0.282
**Steady sexual partner types (** ***n*** ** = 420)**									
Student from the same school as the participant	34(60.7)		155(42.6)		Ref.1			——	
Student from a different school than the participant	12(21.4)		112(30.8)		0.49(0.24–0.99)	0.045		——	
Non-student	10(17.9)		97(26.6)		0.47(0.22–0.99)	0.048		——	
**Condom usage during sexual activity with a steady partner in the past year (** ***n*** ** = 420)**									
Never used	25(44.6)		102(28.0)		Ref.1			——	
Occasional used	20(35.7)		142(39.0)		0.58(0.30–1.09)	0.090		——	
Every time used	11(19.6)		120(33.0)		0.37(0.18–0.80)	0.010		——	

^#^VCT, voluntary HIV counseling and testing

The proportion of participating partners who consistently used condoms was 37.48% (229/611). The rate of consistent condom use was 31.19% (131/420) among the 420 participants who had steady sexual partners in the past year. Participants whose types of steady partners included non-students or students from a different school among the participant (*cOR* = 0.47 or 0.49, respectively), and those who used condom consistently during sexual activity with steady partner(s) (*cOR* = 0.37) were less likely to have undergone VCT.

### Multivariable analysis of factors associated with VCT among the participants

Logistic multivariable regression analysis was performed on variables with *P* < 0.2 in the univariable analysis, as well as variables including sex, grade, and province of household registration. Analysis revealed (Table 2) that, compared with participants in the corresponding control group, the proportion of those with non-Zhejiang household registration undergoing VCT increased by 111% (adjusted OR [*aOR*]: 2.11; 95% Confidence Interval [*CI*]: 1.17–3.80), the proportion of participants who had received AIDS-themed lectures or health education courses from the school in the past year increased by 296% (*aOR*: 3.96; 95% *CI*: 1.49–10.50), the proportion of participants who had received HIV risk self-assessment conducted by the school in the past year increased by 131% (*aOR*: 2.31; 95% *CI*: 1.17–4.59), the proportion of participants who had engaged in commercial sex in the past year increased by 98% (*aOR*: 1.98; 95% *CI*: 1.07–3.66), the proportion of participants who were male participants decreased by 63% (*aOR*: 0.37; 95% *CI*: 0.18–0.77), the proportion of participants who used condom consistently during casual sexual activity decreased by 55% (*aOR*: 0.45; 95% *CI*: 0.21–0.97).

## Discussion

Currently, VCT is a widely acknowledged and effective approach to mitigate the spread of HIV, which enables individuals to be aware of their HIV infection status, choose to undergo HIV testing, and assess the risk for HIV infection, while also developing plans to reduce the risk for HIV infection [20]. In this study, 68 out of 611 participants (11.13%) had engaged in casual sex underwent VCT, which was lower than that of research in other countries [15, 21, 22]. With advances in social development and globalization, individuals are more open to and proactive in accepting knowledge and information related to AIDS. Nevertheless, the prevention and control of HIV infection still has many challenges, including social discrimination, lack of resources for HIV/AIDS prevention and treatment, and low treatment adherence [23]. Therefore, there is a need for the continuous promotion of HIV/AIDS awareness and education, strengthening community cooperation, and government support to improve understanding and awareness of HIV/AIDS prevention.

Results of the present study indicated that the proportion of non-Zhejiang residents participating in VCT increased by 111%, which substantiates the expansion and promotion of testing services in Zhejiang Province. This was achieved through various measures, including the establishment of accessible VCT centers, intensified promotion, and educational efforts. The objective was to encourage greater engagement in HIV testing among residents with non-Zhejiang household registration. These measures were part of broader initiatives aimed at increasing VCT uptake among all young students in Zhejiang Province, which promoted the provision of services, making it relatively convenient for university students to access testing services, especially for non-Zhejiang household registration students to participate. Expanded service coverage of VCT and increased efficacy of promotional activities likely contributed to the observed increase in HIV testing rates [24, 25]. Nevertheless, it is essential to acknowledge that the 111% increase in VCT participation does not entirely represent the prevalence of HIV infection among residents with non-Zhejiang household registration. Therefore, additional research is required to enhance the promotion and management of HIV/AIDS prevention.

A comprehensive understanding of the transmission routes and preventive measures of AIDS is essential for both individuals and society [26]. The findings revealed a 296% increase in VCT participation among individuals who engaged in AIDS-themed lectures or school-led health education classes, showcasing the proactive promotion and implementation of AIDS education. Schools’ initiatives in AIDS education primarily encompass professional lectures, training courses, promotional materials, and counseling services [21, 27]. This trend demonstrates students’ awareness of AIDS-related matters and validates the efficacy of school-based AIDS education. The notable improvement in these data holds substantial importance in advancing AIDS prevention and control, as well as safeguarding the well-being and safety of students. The active engagement of students in VCT signifies their eagerness to comprehend their health status and promptly undertake the appropriate measures. This positive attitude indicates the effectiveness of AIDS education and establishes a firm groundwork for preventing and controlling the spread of AIDS.

Findings of this study suggest a 131% increase in the proportion of participants who voluntarily sought VCT after undergoing an HIV risk self-assessment in the past year. HIV risk self-assessment increases awareness of the risk for HIV infection and encourages HIV testing [28]. Active participation in these activities enables students to better identify the potential HIV risks that they may encounter, thereby fostering voluntary engagement in VCT. A prospective controlled study involving healthcare students and teenagers found that peer educators focusing on sexual and reproductive health (SRH) showed a significantly greater improvement in SRH knowledge scores compared to their peers. Furthermore, adolescents engaged in public peer-led health prevention programs demonstrated a more pronounced enhancement in their SRH knowledge scores than their counterparts [29]. Future efforts should focus on strengthening the promotion of AIDS education and risk self-assessment activities among student groups. By enhancing awareness and knowledge of HIV/AIDS, students will be more inclined to proactively undergo VCT to safeguard their health.

Commercial sexual activity often involves sexual practices among partners for whom infection status is unknown, thereby increasing the risk for STDs [30]. The study results indicated a 98% increase in the proportion of participants who engaged in commercial sexual activity in the past year and underwent VCT, compared with the group that did not engage in commercial sexual activity. This increase may reflect the participants’ improved understanding of the importance of sexual health and education. Individuals involved in commercial sexual activity may possess better knowledge of sexual health and infectious diseases, as well as the importance of regular sexual health check-ups [31, 32]. This heightened awareness will play a crucial role in enhancing sexual health and curbing STD transmission. Failure to take the necessary safety precautions or engage in sexual relationships with partners whose HIV infection status is unknown may increase their susceptibility to infection [15, 27]. Hence, an increase in the proportion of individuals engaged in commercial sexual activity who undergo VCT can also be considered a protective measure to detect and manage early HIV infection. This finding underscores the importance of enhancing sexual health education and ensuring accessible VCT services to foster healthier sexual behaviors and control transmission of STDs.

The study findings suggested a 63% decrease in the proportion of male participants in the VCT group compared with the female student group. This increase suggests a heightened focus on self-protection and health among female students. With the growing awareness and understanding of HIV/AIDS, female students are becoming more aware of their own risks and responsibilities, and are actively seeking VCT services to protect their own and others’ health. Numerous non-governmental organizations across various countries advocate and facilitate women’s engagement in VCT services through various measures, including expanding opportunities and resources, and enhancing support and protection for women [33]. Concurrently, there is a need to augment male students’ involvement in VCT services, which encompasses enhancing education, reinforcing resource allocation, and fostering a more inclusive and supportive environment in social, cultural, and economic terms.

The use of condoms is widely recognized as an effective method for reducing the risk for contracting STDs, and the failure to consistently use condoms during casual sexual encounters increases the risk for transmission of STDs [34]. Findings from this study revealed that compared to those who never utilized condoms, there was a 55% reduction in the proportion of individuals who consistently used condoms for VCT during casual sexual encounters. Consequently, this reduction in proportion can be interpreted as an indication of behavioral change and a decline in awareness of sexual safety. This could be attributed to either a diminished perception of STD risk among participants or a lack of awareness regarding the significance of the VCT [27]. Consequently, it is imperative to enhance health education and promote endeavors to augment individuals’ understanding of the significance of practicing safe sex and consistently using condoms during sexual activities.

However, the implementation of VCT activities among young students faces challenges and influencing factors. First, there is a lack of comprehensive sexual education. In many schools and universities, the content and coverage of sex education are very limited, resulting in students engaging in risky sexual activity with inadequate knowledge of the risks and prevention measures for HIV transmission [35]. Even if VCT services are available within or near school campuses, these students may be unaware of their importance, leading to lower enthusiasm for and participation rates in VCT. Additionally, social and cultural beliefs may have influenced these results. In some societal and cultural environments, discussing sexual behavior and HIV may be regarded as indecent or “taboo”, and could also trigger moral controversies. This discourages students from actively seeking VCT. Some students may choose to remain silent and not seek HIV-related healthcare and counseling services out of fear of discrimination or stigmatization. Additionally, the accessibility of health services cannot be overlooked in terms of their impact on VCT. Some schools and universities lack specialized health facilities, making it difficult for students to access HIV testing and counseling services. If students incur high costs or time undergoing testing, this can reduce their likelihood of participating in VCT activities.

The present study had several limitations, the first of which was its cross-sectional survey design, which precluded the establishment of causal relationships between the factors explored. The potential impact of social desirability bias on self-reported behaviors and attitudes towards VCT and sexual practices would affect the results in this study. Additionally, patients may have experienced recall bias while completing the questionnaires, potentially introducing bias into the results. However, we performed multiple regression analysis to account for confounding variables and minimize systematic errors attributable to individual factors. Moreover, certain variables in the study contained missing values, which may have introduced a degree of bias in the study population. We removed missing variables or classified them separately without changing the results of the multivariate statistical analysis. Given the insignificance of the proportion of missing values in these variables, we excluded them before performing statistical analyses and relevant calculations. However, we did not perform a comparative analysis of awareness of AIDS prevention and control knowledge among students from different majors in this study. For example, students studying medicine or biology may be more aware than study samples in the literature, which may have affected the results. Owing to China’s vast geography and significant differences in socioeconomic levels, and although this survey selected Zhejiang Province as the research region, there may be certain differences when extrapolated to the national level. As such, additional multi-center prospective randomized cohort studies are necessary to validate our findings.

## Conclusions

Findings of the present study suggested that college students who engaged in casual sexual activity were more likely to participate in VCT if they were non-Zhejiang residents, attended HIV/AIDS lectures or health education classes within the past year, underwent HIV risk self-assessment, or engaged in commercial sexual activity. Conversely, male students and those who consistently used condoms during casual sexual encounters were less likely to participate in VCT. These findings provide valuable insights for the development of effective HIV prevention and control strategies for young students, highlighting the importance of strengthening VCT promotion and outreach efforts among non-Zhejiang residents, students engaged in commercial sexual activity, and those who have not received HIV/AIDS education. Furthermore, it is crucial to undertake additional research and implement HIV behavioral interventions specifically tailored to young(er) students. This will enhance the awareness of HIV infection and promote safer practices.

## Data Availability

No datasets were generated or analysed during the current study.

## Abbreviations

95% *CI*: 95% confidence interval
HIV: Human immunodeficiency virus
AIDS: Acquired immunodeficiency syndrome
CNY: Chinese Yuan
STDs: Sexually transmitted diseases
VCT: voluntary counseling and testing
SRH: Sexual and reproductive health
